# Weighted Gene Co-Expression Network Analysis Based on Stimulation by Lipopolysaccharides and Polyinosinic:polycytidylic Acid Provides a Core Set of Genes for Understanding Hemolymph Immune Response Mechanisms of *Amphioctopus fangsiao*

**DOI:** 10.3390/ani14010080

**Published:** 2023-12-25

**Authors:** Yongjie Wang, Xipan Chen, Xiaohui Xu, Jianmin Yang, Xiumei Liu, Guohua Sun, Zan Li

**Affiliations:** 1 School of Agriculture, Ludong University, Yantai 264025, China; 2 College of Life Sciences, Yantai University, Yantai 264005, China; xiumei0210@163.com

**Keywords:** *Amphioctopus fangsiao*, hemolymph, LPS, Poly I:C, WGCNA, protein–protein interaction networks, immune response

## Abstract

**Simple Summary:**

The health of *Amphioctopus fangsiao*, a species used in aquaculture or fish farming, can be greatly affected by infections. To understand how their immune system responds, we looked at the changes in their immune cells when exposed to substances that mimic these infections. By studying these responses, we were able to identify key parts of their immune system that change when faced with infection-causing threats. We also discovered important genes, including *PKMYT1* (protein kinase, membrane associated tyrosine/threonine 1) and *NAMPT* (nicotinamide phosphoribosyltransferase), that play a crucial role in this response. Our study gives us a deeper understanding of the immune system of the *Amphioctopus fangsiao*.

**Abstract:**

The primary influencer of aquaculture quality in *Amphioctopus fangsiao* is pathogen infection. Both lipopolysaccharides (LPS) and polyinosinic:polycytidylic acid (Poly I:C) are recognized by the pattern recognition receptor (PRR) within immune cells, a system that frequently serves to emulate pathogen invasion. Hemolymph, which functions as a transport mechanism for immune cells, offers vital transcriptome information when *A. fangsiao* is exposed to pathogens, thereby contributing to our comprehension of the species’ immune biological mechanisms. In this study, we conducted analyses of transcript profiles under the influence of LPS and Poly I:C within a 24 h period. Concurrently, we developed a Weighted Gene Co-expression Network Analysis (WGCNA) to identify key modules and genes. Further, we carried out Gene Ontology (GO) and Kyoto Encyclopedia of Genes and Genomes (KEGG) enrichment analyses to investigate the primary modular functions. Co-expression network analyses unveiled a series of immune response processes following pathogen stress, identifying several key modules and hub genes, including *PKMYT1* and *NAMPT*. The invaluable genetic resources provided by our results aid our understanding of the immune response in *A. fangsiao* hemolymph and will further our exploration of the molecular mechanisms of pathogen infection in mollusks.

## 1. Introduction

*Amphioctopus fangsiao*, an economically important species in China’s coastal areas, has attracted attention due to the quality of its meat and its high medicinal value [1,2]. However, bacterial and viral outbreaks during its cultivation often result in substantial economic losses [3]. Aquatic pathogen infections, including common viruses (ISKNV, IHNV) and bacteria (*Vibrio vulnificus*, *Vibrio anguillarum*), are extensively reported in marine organisms, causing severe infectious diseases [4,5,6,7,8]. In response to such invasions, the immune system mounts a robust defense that ensures the organism’s safety by identifying and eliminating the invading pathogens [9,10]. Hemolymph, a primary mover of immune cells, plays a crucial role in the mollusk immune system by facilitating rapid and targeted immune responses throughout the organism [11,12]. Pathogens that cause high rates of disease in marine aquaculture in *A. fangsiao* include *Vibrio anguillarum*, *Vibrio Parahemolyticus*, and *Edwardsiella tarda*. Infected *A. fangsiao* can show varying degrees of tissue damage, leading to increased culture mortality [13,14,15]. Lipopolysaccharides (LPS) and polyinosinic-polycytidylic acid (Poly I:C), both recognized by pattern recognition receptors (PRRs), are typically employed to mimic bacterial and viral invasions, respectively [16,17]. The stimulation of *A. fangsiao* with LPS and Poly I:C, followed by transcriptome analysis of its hemolymph, will provide deep insights into the molecular immune mechanisms of *A. fangsiao*.

The advancement of high-throughput sequencing technology and data analysis methods in recent years has facilitated the discovery of numerous immune-related genes in invertebrates [18,19,20]. These extensive datasets significantly contribute to our knowledge of specific immune responses. Weighted Gene Co-expression Network Analysis (WGCNA), an instrumental bioinformatics tool, aids in understanding gene functions and associations. It enables identification of the correlation among genes with analogous expression patterns [21,22]. One of the main benefits of WGCNA is transforming gene expression data into modules. By analyzing these modules related to traits as a unit, it minimizes computational requirements and enhances accuracy [23,24]. However, the application of WGCNA in invertebrate immunity is scarcely documented.

This study encompasses the analysis of the transcription profiles of hemolymph tissues at 6 and 24 h under two distinct types of stimulation [25,26]. Employing the union of differentially expressed genes (DEGs) within a 24 h period under both stimulation types, the WGC network was constructed. Furthermore, the study utilized GO and KEGG functional enrichment analyses to deduce the relationships between co-expressed genes and immunity. A protein–protein interaction network was also built to explore the functional relationships of key genes between modules. Lastly, the expression patterns of 19 key genes were screened and corroborated using quantitative RT-PCR. The results procured from this research provide a valuable resource and crucial foundation for future studies on the immune mechanism of *A. fangsiao*.

## 2. Materials and Methods

### 2.1. Experimental Procedure

The above experimental procedure and raw data obtained by sequencing were derived from Chen et al. [25,26].

Healthy *A. fangsiao* were categorized into four groups. The LPS group consisted of 40 animals treated with an intramuscular injection of 100 µL LPS solution, the Poly I:C group also had 40 animals, which were subjected to an intramuscular injection of 100 µL Poly I:C solution, the PBS group consisted of 40 animals treated with an intramuscular injection of 100 µL PBS solution. Lastly, the blank control group contained 20 untreated animals. Additionally, nine *A. fangsiao* were randomly selected as blank control before injection. Hemolymph cells were swiftly gathered in Trizol and preserved in liquid nitrogen. PBS, Poly IC, and LPS were applied for stimulation experiments with hemolymph tissue extracted at the 6 h and 24 h post-injection mark.

Total RNA was analyzed using Trizol reagent in compliance with the manufacturer’s instructions. Quality control of RNA was performed by Agilent 2100 bioanalyzer.

For RNA extraction, nine *A. fangsiao* were randomly picked from each time point group: *A. fangsiao* injected at 0 h (B0h), *A. fangsiao* injected with PBS at 6 h (P6h), *A. fangsiao* injected with PBS at 24 h (P24h), *A. fangsiao* injected with Poly I:C at 6 h (IC6h), *A. fangsiao* injected with Poly I:C at 24 h (IC24h), *A. fangsiao* injected with LPS at 6 h (L6h), and *A. fangsiao* injected with LPS at 24 h (L24h). For each group, nine *A. fangsiao* were randomly selected and divided into three subgroups. The RNA of the same mole ratio from each *A. fangsiao* subset was combined to create templates for RNA-Seq library construction. The library preparations were sequenced on an Illumina Novaseq platform and 150 bp paired-end reads were generated. The residual hemolymph RNA post-library construction was utilized for quantitative affirmation via quantitative real-time PCR.

The SRA number for the NCBI database is: SRR20338038–SRR20338058. All subsequent analyses based on sequencing data obtained by Chen et al. are completely new research.

### 2.2. Identification of DEGs

The initial step in ensuring the accuracy of subsequent analyses requires careful screening of the raw data. This includes the removal of reads containing adapters, unknown base information, and reads of subpar quality. Following this, Trinity software (v2.6.6) is employed to assemble the clean reads into a reference sequence; the sequence cluster of the highest length is then selected by Corset for further analysis. The reference sequence analyzed by Trinity and used as the transcriptome maps the clean reads of each sample. Later, the mapping process is facilitated by RSEM software (v1.3.3) using default parameters. The FPKM values, signifying expression levels, are computed for each gene based on read counts and the gene length. In order to screen out differentially expressed genes between various treatment groups, the DESeq2 R package (1.20.0) is utilized. Gene selection is determined based on a *p*-value ≤ 0.01 and a |log2fold change| ≥ 1 and categorized as DEGs.

### 2.3. Bioinformatics Analysis

In this study, 561 DEGs were identified between the experimental and control groups six hours after LPS injection. At the 24 h mark, there were 778 DEGs observed. Similarly, after the injection of Poly I:C, 1082 and 299 DEGs were discovered at the six and 24 h intervals between both groups, respectively. Through sequencing, the analysis unearthed 21 RNA-Seq datasets. The expression data from 2343 genes—representing the union set of DEGs—from all four groups were subsequently selected for the WGCNA. In order to establish a correlation among genes that adheres to scale-free topology network criteria, the most suitable power *β* was selected [27]. This was achieved when the power was at its minimum, yet maintaining a R^2^ value above 0.85. Furthermore, a hierarchical clustering tree was constructed utilizing the correlation coefficients between genes. Consequently, genes with similar expression patterns were grouped into the same module. Those not assigned were designated into the grey module. The applied thresholds for module clustering were a minimum module size of 30 and cutting height of 0.75.

### 2.4. Functional Analysis of Module Genes and Identification of Hub Genes

In this study, we assessed the correlation coefficients between modules and traits using eigenvalues, identifying the ones with the highest correlation to trait eigenvalues as key modules. We conducted an analysis of the primary functions of modular genes using GO and KEGG enrichment. Within these modules, genes exhibiting high connectivity are considered to bear significant biological implications. To further narrow down this investigation, we designated 20 genes from each key module and earmarked the top three genes demonstrating the greatest connectivity as hub genes. Utilizing Cytoscape, we visualized the interactions of hub genes. The tool String (https://cn.string-db.org/, accessed on 4 August 2022) facilitated the construction of protein interaction networks, providing insight into the relationships among genes in distinct modules. From this pool, we identified six hub genes with greater numbers of protein interaction.

### 2.5. qPCR Validation

We isolated 19 hub genes for validation using qRT-PCR. Primer Premier 5.0 facilitated the creation of gene-specific primer sequences, the details of which are found in Table 1. We evaluated the stability of *GAPDH*, *β-ACTIN*, and *18 S* genes across varying tissues and developmental stages of *A. fangsiao*. Due to its relatively stable expression levels at all measured time points, we selected *β-ACTIN* as the reference gene. The fluorometric quantification procedure adhered to the method previously outlined by Liu et al. [28].

## 3. Results

### 3.1. Identification of DEGs

In this investigation, upon LPS stimulation, we identified 561 and 778 DGEs at the 6 h and 24 h marks, respectively. Post-Poly I:C stimulation, 1082 and 299 DGEs were pinpointed at 6 h and 24 h, respectively. For future analyses, we collated these observations into a union of the four groups of DGEs, encompassing 2343 genes. After rigorous data quality control, the average Q20 and Q30 of clean reads exceeded 90%, which proved that the sequencing results were qualified and satisfied the subsequent analysis [27,28]. The read length of each sample as well as the mapping rate is shown in the attached table (Table S1). The reference genome is derived from the splicing of clean reads.

### 3.2. Weighted Gene Co-Expression Network Analysis

**Figure 1:**
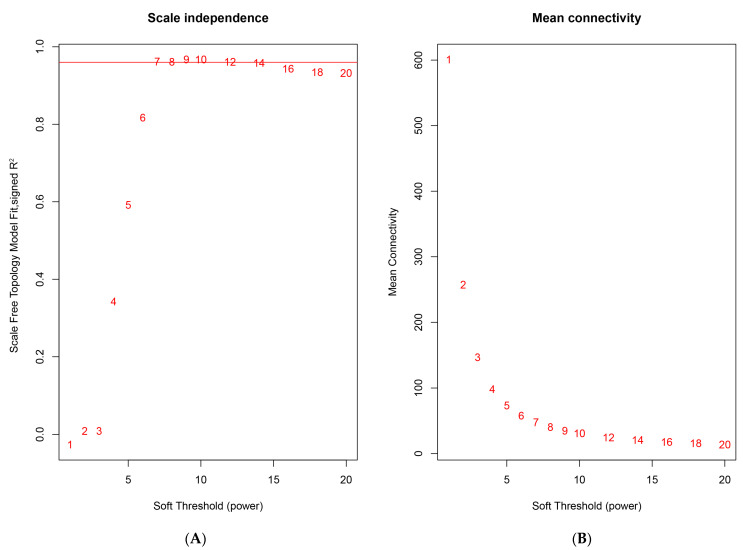
Diagram of power value and connectivity. (**A**) The *x*-axis delineates the power *β*, with the *y*-axis symbolizing R^2^. The line in red signifies R^2^ = 0.96. (**B**) The *x*-axis marks the power *β*, and the *y*-axis portrays the mean connectivity.

**Figure 2:**
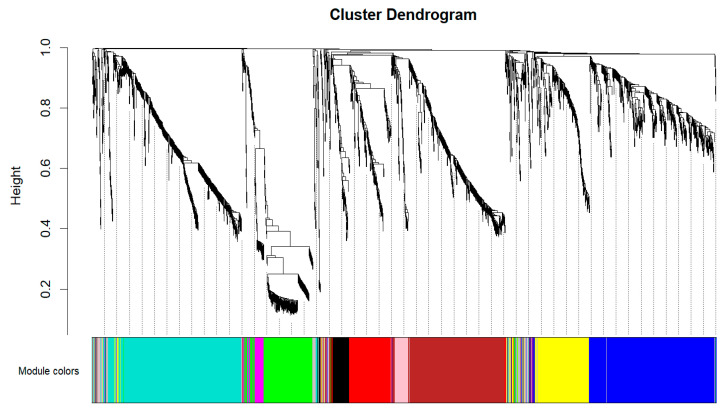
Hierarchical cluster tree of DEGs. Each node of the tree represents a DEG. The associated color denotes its corresponding module. The grey module encompasses all unclustered DEGs.

Weighted correlation network analysis is a systems biology approach used to characterize patterns of genetic associations between different samples. It is usually biologically meaningful to choose the smallest soft threshold for the square of the correlation coefficient (R^2^) greater than 0.85 as the power value for subsequent analysis. The WGCNA was performed on 2343 DEGs, selecting a power of 7, the minimum power where the square of the correlation coefficient (R^2^) exceeded 0.85, ensuring high biological significance of the network (Figure 1A). Concurrently, average connectivities of DEGs underwent evaluation across different power *β* values (Figure 1B). Ten distinct modules were identified and clustered (Figure 2), with sizes ranging from 53 to 515 (Table 2).

### 3.3. Screening and Functional Analysis of Key Modules

**Figure 3:**
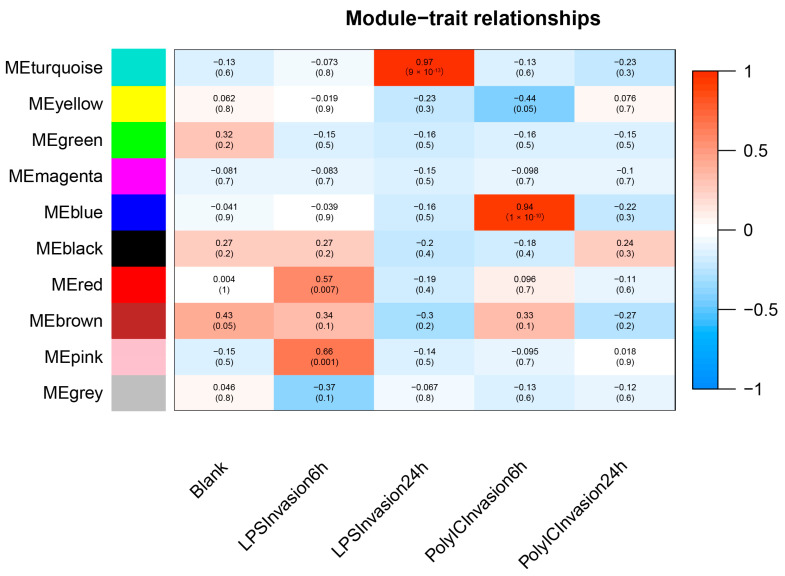
The diagram presents relationships between modules and traits. Each row represents a module, while each column depicts a trait. The significance levels are visually displayed using a color spectrum that ranges from blue, indicative of low significance, to red, denoting high significance.

**Figure 4:**
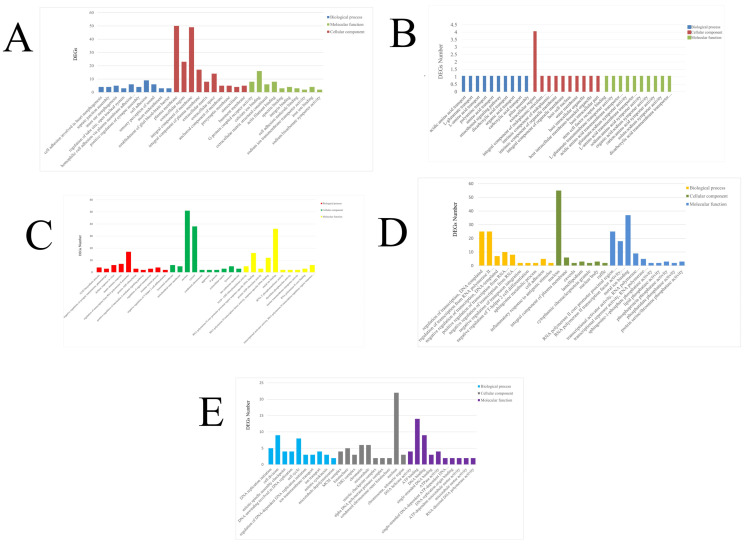
Level-3 GO terms of five modules. (**A**) GO terms enriched with DEGs in the brown module. (**B**) GO terms enriched with DEGs in the pink module. (**C**) GO terms enriched with DEGs in the turquoise module. (**D**) GO terms enriched with DEGs in the blue module. (**E**) GO terms enriched with DEGs in the black module.

**Figure 5:**
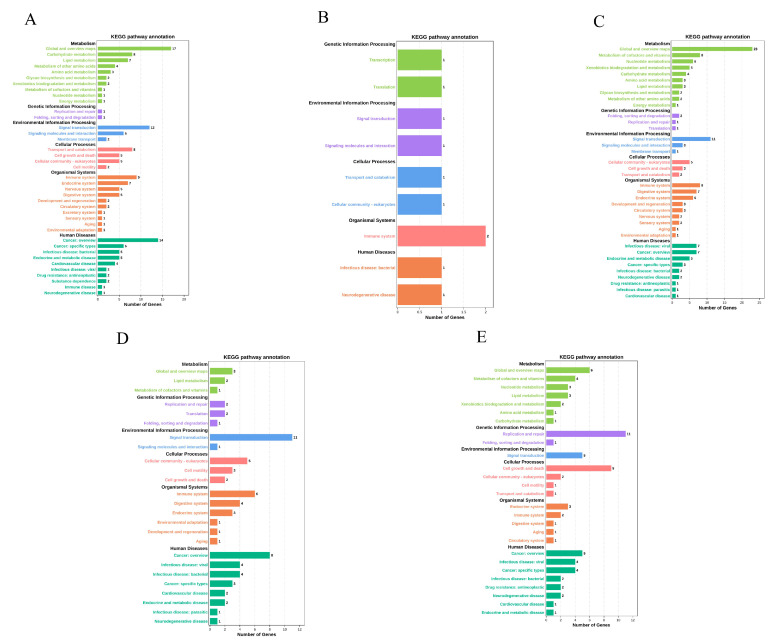
Level-2 KEGG terms of five modules. (**A**) KEGG terms enriched with DEGs in the brown module. (**B**) KEGG terms enriched with DEGs in the pink module. (**C**) KEGG terms enriched with DEGs in the turquoise module. (**D**) KEGG terms enriched with DEGs in the blue module. (**E**) KEGG terms enriched with DEGs in the black module.

This study identified five modules as related to stimulation time, as determined by the eigenvalues of each module. Of these, there was a significant correlation between the brown, pink, and turquoise modules and the LPS invasion time, as well as between the brown, blue, and black modules and the Poly I:C invasion time (Figure 3). Subsequent GO and KEGG enrichment analyses explored the function of each module. The brown module was enriched with 195 GO subclasses (biological process, cellular component, and molecular function), and the pink, turquoise, blue, and black modules were enriched with 223, 153, 67, and 77 GO subclasses, respectively. Figure 4 presents the top 10 GO level-3 terms in these three categories. Several immune-related terms, including cell surface receptor signaling pathway, defense response to virus, innate immune response, positive regulation of interleukin-4-mediated signaling pathway, immune system process, negative regulation of interferon-gamma-mediated signaling pathway, inflammatory response to antigenic stimulus, chemotaxis, and regulation of the apoptotic process, were significantly enriched. Similarly, in level-2 KEGG terms, immune system, infectious disease: bacterial, infectious disease: viral and parasitic were significantly enriched (Figure 5).

### 3.4. Construction of Key Networks and Identification of Hub Genes

**Figure 6:**
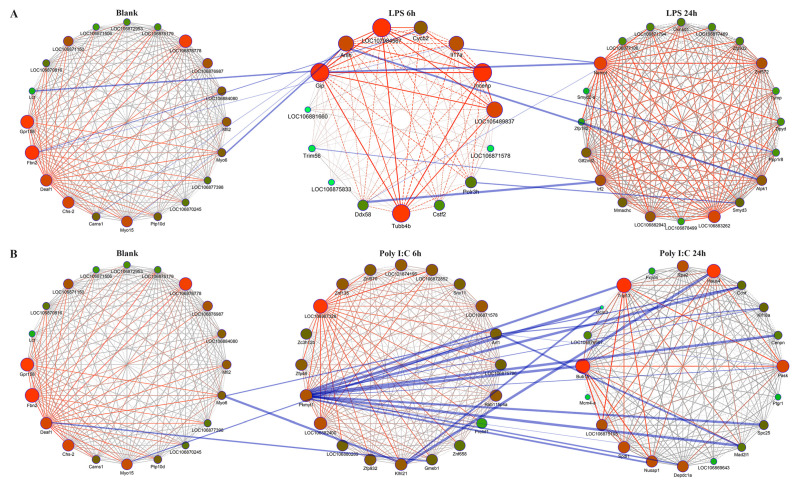
Gene interaction networks. (**A**) Gene networks are depicted at various time intervals following LPS stimulation. Each circle in the diagram represents a unique gene. The color gradient of the circle, transitioning from green to red, coupled with the transition of the circle size from small to large, symbolizes the gene connectivity from low to high. Within an individual module, a transition of line colors from dark to light and a shift of line thickness from thin to thick represent gene correlations from low to high, respectively. Furthermore, the lines interconnecting various modules signify the interactional relationships between the genes. (**B**) Gene networks at different time intervals following Poly I:C stimulation are also depicted.

We have constructed gene networks pertinent to the LPS invasion timeline from three modules, utilizing the 20 key genes present in each. This is barring the pink module. Concurrently, a protein–protein interaction network was assembled to aid in discerning the functional connections between the key genes originating from various modules (Figure 6A). Among these genes, *DEAF1*, *FBN2*, and *GPR158* exhibited the highest connectivity in the brown module and were therefore categorized as hub genes. Similarly, *GIP*, *INCENP*, and *LOC107984567* were identified as hub genes due to their high connectivity in the pink module, while *NAMPT*, *LOC106883262*, and *ZNF572* were highlighted in the turquoise module. An additional protein–protein interaction network was crafted to explore the immune relationships across the three modules. Three genes—*ANLN*, *NAMPT*, and *IRF2*—were classified as hub genes (Table 2). Notably, *NAMPT* displayed high connectivity and was found to bear close relationships to the key genes from the other modules.

Moreover, we built gene networks related to the Poly I:C invasion timeline, again employing the 20 key genes in each module. The blue, brown and black modules were considered. Similar to earlier procedure, another protein–protein interaction network was assembled to explore functional relationships of the key genes across modules (Figure 6B). Here, *DEAF1*, *FBN2*, and *GPR158* were identified as hub genes in the brown module due to their high connectivity. Likewise, *LOC106867328*, *LOC106882400*, and *PKMYT1* from the blue module, along with TRIP13, BUB1B, and HAUS4 from the black module, were recognized as hub genes. In an extension of the previous investigation, we constructed another protein–protein interaction network to assess the immune relationships between the three modules and identified *PKMYT1*, *ARF1*, and *KLHL2* as the hub genes (Table 3). Notably, *PKMYT1* demonstrated high connectivity and appeared to be closely related to the key genes of the other modules.

### 3.5. Quantitative RT-PCR Validation of Hub Genes

**Figure 7:**
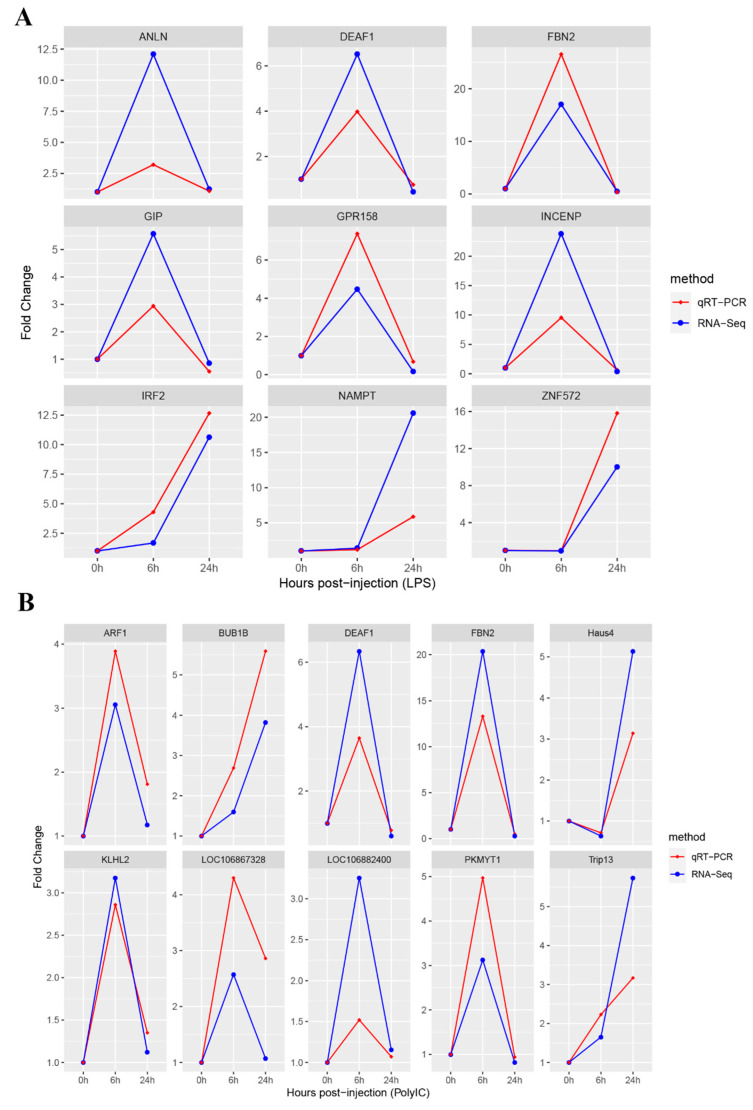
Comparison of expression of hub genes between qRT-PCR and RNA-Seq results. (**A**) qRT-PCR validation results of genes related to LPS infection. The *x*-axis stands for the infection time; the *y*-axis represents fold change. (**B**) qRT-PCR validation results of genes related to Poly I:C infection.

The sum of the top three highest scoring genes in each of the most relevant modules and the three most important genes in each gene interaction network totaled 19 genes for validation. The expression levels of the 19 identified hub DEGs were evaluated at each time point using qRT-PCR. Subsequently, these expression levels were juxtaposed against those ascertained from RNA-Seq. By conducting a correlation analysis of all qRT-PCR results with the RNA-Seq data, it was observed that similar expression patterns emerged consistently. The resulting trends are illustrated in Figure 7.

## 4. Discussion

### 4.1. The Intention of this Study

*A. fangsiao*, a significant economic species prevalent in the coastal waters of China, possesses both high nutritional and medicinal value [2]. However, in the culture process, *A. fangsiao* is vulnerable to infections caused by pathogenic microorganisms such as bacteria, viruses, and parasites. These infections often lead to widespread diseases, resulting in substantial economic losses [29,30]. The integrity of the hemolymph is critical for the health of *A. fangsiao*, due to its significant role in promoting organism defense through immune processes [12]. Understanding the alterations in the hemolymph transcript profile following pathogen invasion may expedite our exploration of *A. fangsiao*’s immune molecular mechanisms. This study delves into the hemolymph immune response mechanisms triggered within 24 h upon stimulation by LPS and Poly I:C.

### 4.2. Discussion of Key Modules and Hub Genes

The research utilized WGCNA on a union set of four groups containing 2343 DGEs. This approach affords a novel insight into the immune molecular mechanisms of *A. fangsiao*. Subsequently, we identified five modules, each associated with a different time-point following two types of stimulation. We employed GO and KEGG enrichment analyses to explore the functionality of these modules. Notably, we discovered a substantial enrichment of immune-related terms, such as: cell surface receptor signaling pathway, defense response to viruses, innate immune response, positive regulation of interleukin-4-mediated signaling pathway, immune system process, negative regulation of interferon-gamma-mediated signaling pathway, inflammatory response to antigenic stimuli, chemotaxis, and regulation of the apoptotic process. We then screened three hub genes from each model, based on superior intramodule connectivity, and identified six additional hub genes considering their high protein interaction numbers. We hypothesize that the above genes play a significant role in the immune response of *A. fangsiao* following pathogen-induced stress.

#### 4.2.1. Analysis of Hub Genes in the Brown Module Associated with 0 h Stimulation

In this study, the hub genes *DEAF1*, *FBN2*, and *GPR158* were identified and found to be enriched in the brown module. Deformed Epidermal Autoregulatory Factor 1 (DEAF1) is a transcription factor associated with autoimmune disorders and has been previously found to bind TTCG motifs [31,32]. Its role as an immunoregulatory factor has been extensively studied in Drosophila [33]. Serving as a cofactor for immune-regulated genes, *DEAF1* is capable of adjusting the expression level of immune response genes [34], with its upregulation preventing adverse consequences caused by excessive immune genes expression. *FBN2* is an integral component of the extracellular matrix (ECM) that moderates the early stages of elastic fiber assembly [35]. The ECM is highly organized and capable of integrating complex signals to immune cells in a spatially patterned and regulatory manner, notably impacting the behavior of leukocytes within inflamed tissues [36]. Importantly, *FBN2* has been found to be associated with immune system diseases as a single gene [37]. It was postulated that *FBN2* has an immune function as a matrix signal, influencing the immune responses of hemolymph. G-protein-coupled receptors (GPCRs), including the orphan *GPR158* of the GPCR family C, have been implicated in various physiological and disease processes [38]. *GPR158* is known to recruit the regulator of G protein signaling (RGS), thereby controlling the activity of other GPCRs [39]. More importantly, GPCR signaling is critical for the spatiotemporal control of leukocyte dynamics during immune responses [40]. These genes potentially have a significant influence on normal immune responses, and their mechanisms of immunoregulation in *A. fangsiao* warrant further investigation.

#### 4.2.2. Analysis of Hub Genes in the Pink Module Associated with 6 h LPS Stimulation

The hub genes *GIP*, *INCENP*, and *LOC107984567* were found to be enriched in the Pink module. The glucose-dependent insulinotropic peptide (GIP) is known to regulate glucose metabolism under inflammatory conditions and stimulate the secretion of inflammatory cytokines and chemokines [41,42]. Various immune cell subsets have been found to express receptors for GIP, which signifies the immune system as another field for incretin action [43]. Recent findings suggest that *GIP* has a direct influence on myeloid immune cells in managing inflammation [44]. These findings led us to hypothesize that *GIP* plays a crucial role in initiating and managing the inflammatory response post-LPS stimulation. The chromosomal passenger complex protein *INCENP* is necessary for chromosome condensation, spindle attachment and function, and cytokinesis during mitosis [45]. It is known that immunosuppression often occurs through cell cycle arrest, a standard procedure adopted by immunotoxins. *INCENP*, being closely related to cell proliferation cycles and tissue repair, has been implicated as candidate genes indicating proliferative effects in immune cells [46,47]. Our results suggest that *INCENP* could be involved in the proliferation of immune cells to sustain immunity. In this study, we propose that *LOC107984567* has a significant relation to the immune response of *A. fangsiao* due to its high connectivity. The functional analysis of other genes within this module suggests that *LOC107984567* might play a role in the regulation of inflammatory responses or cell proliferation. All three genes were noticeably upregulated at the 6 h mark following LPS stimulation. However, the functions and interconnections of these genes in *A. fangsiao* have not been documented and warrant further research.

#### 4.2.3. Analysis of Hub Genes in the Turquoise Module Associated with 24 h LPS Stimulation

The turquoise module disclosed a significant amount of DEGs, hinting at the potential presence of extensive immune responses within this module. Central to the module are hub genes such as *NAMPT*, *LOC106883262*, and *ZNF572*, which are hypothesized to contribute to immune response processes as primal regulatory elements. Recognized as a member of the nicotinic acid phosphoribosyltransferase (NAPRTase) family, *NAMPT* is implicated in a multitude of crucial biological undertakings, spanning metabolism, stress response, and aging [48]. Beyond its enzymatic activity, *NANMPT* operates as a cytokine, significantly influencing immune response regulation [49]. Its demonstrated involvement in apoptosis regulation further extends to inhibiting apoptosis instigated by various inflammatory responses [50]. *NAMPT* is also perceived as a novel intermediary of innate immunity [51]. As for Zinc-finger (ZNF) proteins, they are known for nucleic acid binding and contributing significantly to vital cellular functions, which include cell proliferation, differentiation, and apoptosis [52]. Recent findings have highlighted the use of *ZNF572* as a potential marker for oxidative stress and infections [53], fostering the assumption that *ZNF572* might play a pivotal role in immune functionality within *A. fangsiao* through transcription regulation. In the context of *LOC106883262*’s high connectivity within the module, and aligning this with the functional analysis of other hub genes, it is speculated that the *LOC106883262* gene could be profoundly influential in innate immunity. However, a thorough exploration and understanding of its specific mechanism of action is required. These three genes significantly increased their expression 24 h post-LPS stimulation, suggesting their vital role in *A. fangsiao*’s immune response.

#### 4.2.4. Analysis of Hub Genes in the Blue Module Associated with 6 h Poly I:C Stimulation

The blue module revealed three hub genes: *LOC106867328*, *LOC106882400*, and *PKMYT1*. *PKMYT1* encodes a member of the serine/threonine protein kinase family, a key regulator of cell cycle, and an integral part of DNA damage repair (DDR)-related signaling [54]. Foreseeably, perturbations in cell cycle correlate with an accumulating progression of DNA damages and trigger apoptosis [55]. Recent studies have suggested that *PKMYT1* can potentially activate the Notch signaling pathway, implying a possible range of immune functions [56]. This broadens *PKMYT1*’s role in apoptosis response and augments the immune response under viral stress. Notably, these three genes demonstrated a significant upregulation 6 h post-Poly I:C stimulation with high connectivity. This suggests that they might be instrumental in *A. fangsiao*’s response to viral stress. These genes have not been extensively researched in mollusks, warranting further exploration of their functions.

#### 4.2.5. Analysis of Hub Genes in the Black Module Associated with 24 h Poly I:C Stimulation

The three hub genes *TRIP13*, *BUB1B*, and *HAUS4*, associated with stress immunity, may be implicated in diverse immune response processes. DNA damage repair is a primary factor influencing cell survival and death, often triggering cell apoptosis [57]. *TRIP13* has an essential role in both meiosis and mitosis, primarily in DNA damage repair [58]. The protein complex known as shieldin can protect the broken DNA ends and govern the DNA repair mechanism [59]. Notably, shieldin’s function depends on *TRIP13*, which drives the suitable assembly and disassembly of shieldin components [60]. On this ground, we propose that *TRIP13* may participate in cell apoptosis regulation via shieldin, contributing to immune system stability. *BUB1B*, also referred to as mitotic checkpoint serine/threonine kinase B, belongs to the spindle assembly checkpoint (SAC) protein family [61]. Analogous to *TRIP13*, *BUB1B* is significantly associated with DNA repair, performing overlapping functions in the DNA repair biological process [62]. Furthermore, we discovered that *BUB1B* mutations can generate a considerable apoptotic response [63]. Previous studies have reported that HAUS/augmin subunits help regulate the mitotic spindle assembly [64], and the current research indicates that *HAUS8* positively regulates the RLR-VISA-dependent antiviral signaling pathway. It does so by recruiting the VISA complex, which facilitates the activation of transcription factors IRF3 and NF-κB, thereby activating the IFN-β promoter induced by viral infection [65]. However, there have been few reports on *HAUS4*, and we hypothesize it shares similar immune functions with *HAUS8*, but further research is required to validate this claim. These hub genes primarily play critical roles in apoptosis and other immune functions, effectively mitigating the damage caused by viral infection.

#### 4.2.6. Protein–Protein Interaction Network Analyses

The functional interactions of key genes in protein–protein interaction networks have been explored to identify possible hub genes influencing the immune response processes of *A. fangsiao*. Notably, *ANLN*, *NAMPT*, and *IRF2* were identified as vital players in the immune processes of A. fangsiao within 24 h following LPS stimulation. *ANLN*, which encodes for an actin-binding protein, has a critical role in cell growth, migration, and cytokinesis. Its importance extends to participation in the PI3K/PTEN signaling pathway, crucial in cellular life-death control [66,67]. Additionally, *ANLN*’s interaction with multiple immune cells underscores its indispensable role in the immune system [68,69]. Meanwhile, Interferon regulatory factor 2 (IRF2), a family member of the transcriptional factors, plays an instrumental role in modulating IFN-induced immune responses [70]. Precisely, *IRF2* oversees genes linked to apoptosis and immune regulation, thereby attaining a balanced transcriptional activation of IRFs and preventing overreactions in immune response [71]. Moreover, it regulates *TLR* gene expression, thereby contributing to the innate immune response to infections [72]. *NAMPT* was distinguished for its high protein interaction numbers and intramodule connectivity. Given the evidence presented, we infer it serves a significant role in immune response against bacterial infection.

In a similar vein, *PKMYT1*, *ARF1*, and *KLHL2* were recognized as regulatory forces in the immune process of *A. fangsiao* within 24 h of Poly I:C stimulation. *ARF1* plays a fundamental role in autoimmune disease development, presumably by inhibiting apoptosis in activated immune cells [73]. *ARF1* has been reported to affect humoral immunity directly by adjusting *AMP* gene expression. It is also implicated in an alternative immune regulation mechanism [74,75]. Considering the discussions and outcomes of this research, *ARF1*’s role in *A. fangsiao*’s immune functions warrants further investigation. *KLHL2*, an ubiquitin ligases receptor and a substrate adaptor for ubiquitination [76], has been observed regulating innate immune response, adaptive immune response, and the DNA damage response [77,78,79]. However, the immune response of *A. fangsiao* via ubiquitin against virus leveraged by *KLHL2* remains an area for further exploration. Similarly, *PKMYT1* was identified owing to its high protein interaction numbers and intramodule connectivity. In light of the foregoing discussions, we hold that it plays a significant role in the immune response against viral infections.

#### 4.2.7. Summary of Immune Responses under the Two Forms of Stimulation

Our results present hub genes that play key roles in inflammation regulation, apoptosis and DNA repair related processes. The above biological processes are more related to the immune defense, and in combination with the analysis of other DEGs with important responsibilities, we hypothesized that the immune defense in *A. fangsiao* was significantly activated by LPS and Poly I:C stimulation.

## 5. Conclusions

In this study, we examined the immune response mechanisms of *A. fangsiao* under pathogenic stress, constructing respective co-expression and protein–protein interaction networks. We identified 19 hub genes and validated them through qRT-PCR, hypothesizing that these genes hold a crucial role in the immune system due to their high connectivity or interaction. The results present a basis for further comprehension of the immune response mechanisms.

## Figures and Tables

**Table 1:** List of primers used for qRT-PCR validation.

Gene Name	Forward Primer (5′-3′)	TM (°C)	Reverse Primer (5′-3′)	TM (°C)	Length (bp)
EAF1	TTGTCTCTGGGCCATTTAAG	60	ACTGAGAGCGTCGATTAGTA	60	102
FBN2	GTCTGCAAAGCTGGTTACT	60	GATTGGCTGTGTGGTTGA	60	105
GPR158	GCTCCACTTGCACATTTATC	59	GAGAACCTTCGATCCAGAAC	60	106
GIP	CGCGGACTTCCGAAATAATA	60	GTAACAAAGCTGTCGCAAAG	60	120
INCENP	CGTTAGGCTTGGATCTAGTG	60	CCTGGAGCTGAGAACTTTG	60	135
LOC107984567	CTATGGGCTGTGAAGGAATC	60	CGAACAAACGACCGAAGT	60	165
NAMPT	TTAGGGTGCAGGTATAGGAG	60	GAGAAACCCGCCATTTCA	60	114
LOC106883262	CGCTCTCCGGCTATTTATTT	60	TCTGATGGCGGGTATTCT	60	153
ZNF572	CCTGTGTGAATACGTCTGTG	60	ACTGAGAGCAGCAGTTTAAG	60	128
LOC106867328	CACACTCACACGCAAACA	61	CCACACTCTGCTCACTAAATC	60	103
LOC106882400	GAACGACCAACTTCCTTCTC	60	GTATCTGCTCCCTTATCCATTC	60	111
PKMYT1	GAATCTGGAACCCGACATAAC	60	CGTAGCCTCCAGTTGTATCTA	61	118
TRIP13	GAGTGGTCAATGCTCTTCTG	60	GAGGTGAAGGCAAACCTATG	60	150
BUB1B	GGTAACGGACCTTCTTCAAC	60	GGGAGAGGTCTGTGGATTAT	60	108
HAUS4	CTGGCGATTCTGATGTTTCT	60	GCTTCATCAGTTCCTCTACTTC	60	117
ANLN	CTGTTGCTCCACGTCTTATG	61	GGTCTTGAGCACTACCTTTG	60	126
IRF2	TTCTTCCTCTCTCACCTCAC	60	CCACTCAAGGCCTGAAATAC	60	132
ARF1	TATCCAGACATTTGCCTTCC	60	TAGTGAGGGAGAGAGAGAGA	60	100
KLHL2	GAAACATCAGTCGTCTCTGG	60	CTTGTTACCTCCGCTGTATG	60	153

**Table 2:** Summary of module sizes.

Modules	DEG Numbers
grey	100
blue	508
brown	396
green	223
megenta	53
pink	61
red	170
tuquoise	515
yellow	237
black	80

**Table 3:** List of top three gene based on protein–protein interaction networks analyses.

Gene Abbreviated Name	Gene Official Full Name	Protein Interaction Numbers
LPS stimulation (brown, pink, and turquoise modules)		
ANLN	anillin, actin-binding protein	4
NAMPT	nicotinamide phosphoribosyltransferase	3
IRF2	Interferon regulatory factor 2	2
Poly I:C stimulation (brown, blue, and black modules)		
PKMYT1	protein kinase, membrane associated tyrosine/threonine 1	12
ARF1	ADP ribosylation factor 1	6
KLHL2	kelch like family member 2	3

## Data Availability

The data presented in this study are available in article and supplementary material.

## Supplementary Materials

The following supporting information can be downloaded at: https://www.mdpi.com/article/10.3390/ani14010080/s1, Table S1: Summary of sequencing.
