# Supplementary material for: Weighted Gene Co-Expression Network Analysis Based on Stimulation by Lipopolysaccharides and Polyinosinic:polycytidylic Acid Provides a Core Set of Genes for Understanding Hemolymph Immune Response Mechanisms of Amphioctopus fangsiao
Source: Animals (Basel). 2023 Dec 25;14(1):80. doi: 10.3390/ani14010080 (PMC10778463; doi:10.3390/ani14010080)
Supplement: Supplementary file 1 [file animals-14-00080-s001.zip › animals-2525096-supplementary.pdf]

Table S1. Summary of sequencing.

Time points	Read length (bp)	Raw reads	Clean reads	Q20 (%)	Q30 (%)	Mapped reads ratio (%)
C_0 h_1	150	24,292,617	23,630,634	96.79	91.72	78.77%
C_0 h_2	150	22,797,157	22,053,332	97.23	92.72	77.82%
C_0 h_3	150	20,673,146	19,971,181	97.28	92.74	79.46%
P_6 h_1	150	23,044,830	22,488,624	95.51	89.44	77.59%
P_6 h_2	150	22,837,022	22,112,808	97.06	92.19	77.73%
P_6 h_3	150	20,835,318	20,229,284	97.29	92.71	78.38%
P_24 h_1	150	24,126,457	23,436,697	95.67	89.68	78.27%
P_24 h_2	150	21,211,214	20,803,620	97.03	92.37	75.95%
P_24 h_3	150	22,477,843	21,837,815	97.27	92.69	77.46%
IC_6 h_1	150	21,244,570	20,624,641	97.21	92.52	77.65%
IC_6 h_2	150	23,308,875	22,656,434	97.12	92.32	76.81%
IC_6 h_3	150	20,810,453	20,090,988	97.00	92.23	77.30%
IC_24 h_1	150	23,024,831	22,197,148	97.25	92.62	78.72%
IC_24 h_2	150	22,726,845	21,967,735	97.05	92.38	78.72%
IC_24 h_3	150	23,366,816	22,718,451	95.74	89.81	78.52%

Time points	Read length (bp)	Raw reads	Clean reads	Q20 (%)	Q30 (%)	Mapped reads ratio (%)
C_0h_1	150	24,292,617	23,630,634	96.79	91.72	78.77%
C_0h_2	150	22,797,157	22,053,332	97.23	92.72	77.82%
C_0h_3	150	20,673,146	19,971,181	97.28	92.74	79.46%
P_6h_1	150	23,044,830	22,488,624	95.51	89.44	77.59%
P_6h_2	150	22,837,022	22,112,808	97.06	92.19	77.73%
P_6h_3	150	20,835,318	20,229,284	97.29	92.71	78.38%
P_24h_1	150	24,126,457	23,436,697	95.67	89.68	78.27%
P_24h_2	150	21,211,214	20,803,620	97.03	92.37	75.95%
P_24h_3	150	22,477,843	21,837,815	97.27	92.69	77.46%
L_6h_1	150	23,841,801	23,166,375	97.14	92.48	76.20%
L_6h_2	150	22,643,550	22,018,023	97.22	92.54	79.04%
L_6h_3	150	22,988,872	22,355,728	97.16	92.52	77.86%
L_24h_1	150	24,123,249	23,399,559	96.91	91.96	78.71%
L_24h_2	150	23,479,482	22,699,861	97.49	93.37	78.18%
L_24h_3	150	21,960,245	21,232,312	97.18	92.49	79.28%
